# Racemosol Derivatives and Other Metabolites from *Bauhinia malabarica* Bark with Antibacterial Activity

**DOI:** 10.3390/molecules30214308

**Published:** 2025-11-05

**Authors:** Wanchat Sirisarn, Apisara Somteds, Supachai Jadsadajerm, Sutin Kaennakam, Nuttapon Yodsin, Awat Wisetsai

**Affiliations:** 1 Department of Microbiology, Faculty of Medicine, Kasetsart University, Bangkok 10900, Thailand; wanchat.s@ku.th; 2 Department of Industrial Chemistry, Faculty of Applied Science, King Mongkut’s University of Technology North Bangkok, Bangkok 10800, Thailand; somteds.apisara@gmail.com (A.S.); supachai.j@sci.kmutnb.ac.th (S.J.); 3 Department of Agro-Industrial, Food, and Environmental Technology, Faculty of Applied Science, King Mongkut’s University of Technology North Bangkok (KMUTNB), Bangkok 10800, Thailand; sutin.k@sci.kmutnb.ac.th; 4 Department of Chemistry, Faculty of Science, Silpakorn University, Nakorn Pathom 73000, Thailand; yodsin_n@su.ac.th

**Keywords:** *Bauhinia malabarica*, racemosol derivatives, phenolic compounds, antimicrobial activity, Gram-positive bacteria

## Abstract

Racemosol, a natural phenolic compound, is known for its antimicrobial potential, yet experimental studies remain limited. In this study, two new racemosol derivatives (**4** and **5**) and four known compounds (**1**–**3**, **6**) were isolated from the bark of *Bauhinia malabarica* and structurally elucidated using spectroscopic analyses. Most of isolated compounds exhibited notable activity against Gram-positive bacteria, including *Staphylococcus aureus*, *Bacillus subtilis*, and *Listeria monocytogenes*, while showing limited effects on Gram-negative strains. Racemosol (**1**) and its derivatives (**2**, **4**, and **6**) displayed potent antibacterial activity with MIC values of 0.156–0.625 µg/µL and bactericidal properties confirmed by comparable MBCs. Compound **6** exhibited the highest potency, indicating that specific structural modifications enhance activity. These findings provide new insights into the structure activity relationships of racemosol derivatives and highlight *B. malabarica* as a promising natural source of phenolic antibacterial agents.

## 1. Introduction

*Bauhinia* is a large genus within the Fabaceae–Caesalpinioideae family—comprising nearly 300 species of trees, shrubs, and climbers. Members of this genus are distributed mainly in tropical regions, including Africa, Asia, and South America [1]. *Bauhinia malabarica* is a tropical tree widely distributed throughout Thailand. The young leaves and flowers are edible, with the leaves having a sour taste and traditionally employed in folk medicine for wound healing, as a diuretic, and for the treatment of dysentery and menstrual disorders [1,2,3]. Phytochemical studies have revealed the presence of seven flavonol derivatives in the leaves [2]. Investigations of the roots have led to the identification of racemosol and demethylracemosol, along with their putative biogenetic precursors, preracemosol A and preracemosol B [3,4]. These metabolites have demonstrated notable biological properties, including cytotoxic, antimalarial, and anti-inflammatory activities through inhibition of COX-1 and COX-2.

**Figure 1:**
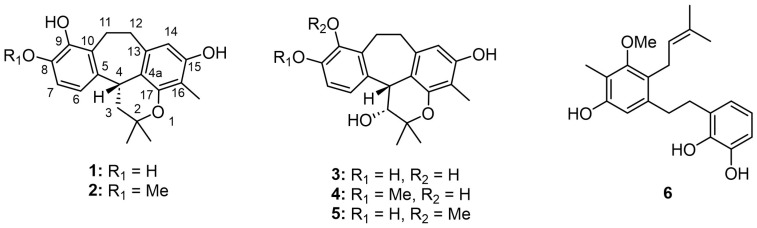
Structures of isolated compounds.

Racemosol—a natural phenolic compound—has been reported to possess notable antimicrobial potential against a broad spectrum of pathogenic microorganisms. To date, the majority of investigations on racemosol have relied predominantly on computational and in silico approaches, particularly in the context of its predicted inhibitory activity against bacteria such as *Mycobacterium tuberculosis [5]*. Experimental studies remain comparatively scarce, with only one report documenting the isolation of racemosol from *B. racemosa*, where it demonstrated activity against *Staphylococcus aureus* and certain *Aspergillus* spp. such as *A. flavus, A. fumigatus* and *A. niger* [6]. Despite the ethnomedicinal importance of the *Bauhinia* genus, which is widely recognized for its diverse secondary metabolites, the clinical relevance of its constituents against pathogenic microorganisms has not been comprehensively established. To address this gap, and as part of our ongoing investigations into bioactive secondary metabolites from natural sources [7], we isolated two new racemosol derivatives (**4**,**5**), along with four known compounds (**1**–**3**, **6**) (Figure 1). The present study describes in detail the isolation and structural elucidation of these compounds and evaluates their antimicrobial efficacy against a broad panel of pathogenic bacteria, thereby providing novel insights into the antimicrobial potential of racemosol derivatives from *B. malabarica*.

## 2. Results and Discussion

Phytochemical investigation of the bark of *B. malabarica* by successive chromatographic separation resulted in the isolation of four known compounds, demethylracemosol (**1**), racemosol (**2**) [3], 3-hydroxy-de-*O*-methyl racemosol (**3**) [8], and preracemosol A (**6**) [3], together with two previously undescribed racemosol derivatives (**4** and **5**). To the best of our knowledge, the structure of compound **2** has previously been determined by NMR and single-crystal X-ray diffraction analysis [9], although the stereochemistry of C-4 was not described. Therefore, we sought to establish the absolute configuration of this stereogenic center. To address this issue, electronic circular dichroism (ECD) calculations were employed.

**Figure 2:**
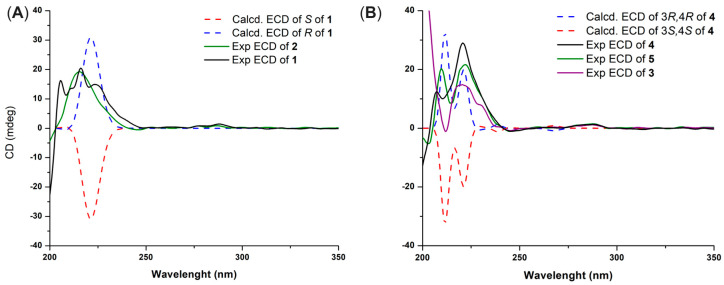
Experimental and calculated ECD spectra of compounds **1** and **2** (**A**) and **3**–**5** (**B**).

The NMR data of demethylracemosol (**1**) and racemosol (**2**) were in complete agreement with those previously reported [9,10]. Compound **1** exhibited optical activity, with [α]_D_^20^ = +16 (*c* 0.10, MeOH). The ECD spectra of the two possible enantiomers were then calculated and compared with the experimental spectrum (Figure 2A). The calculated spectrum for the *R* absolute configuration at C-4 showed good agreement with the experimental data, accordingly, the absolute configuration of **1** was assigned as illustrated in Figure 1. Based on these results, the absolute configuration of racemosol (**2**) was also assigned as *R*, consistent with its observed optical rotation, [α]_D_^20^ = +20 (*c* 0.10, MeOH), and its ECD spectrum (Figure 2A), which exhibited the same Cotton effect sign as that of compound **1**.

3-Hydroxy-8-*O*-methylracemosol (**4**) was obtained as a whitish amorphous solid. HRESIMS showed a molecular ion at *m*/*z* 355.1549 [M–H]^–^ (calcd. for C_21_H_23_O_5_^–^, 355.1551), consistent with the molecular formula C_21_H_24_O_5_. The ^1^H NMR spectrum of **4** (Table 1) displayed a pattern characteristic of the racemosol skeleton, a relatively rare natural product class found in this plant. Two aromatic doublets at *δ*_H_ 6.90 (d, *J* = 8.6 Hz, H-6) and 6.72 (d, *J* = 8.6 Hz, H-7), together with a singlet at *δ*_H_ 6.11 (s, H-14), indicate three aromatic protons. The presence of a pyran ring is confirmed by two methine resonances (one oxygenated and one non-oxygenated) observed at *δ*_H_ 4.33 (overlapping, H-3 and H-4) and two methyl singlets at *δ*_H_ 1.55 (s, Me-5′) and 1.23 (s, Me-6′). Two methylene groups were observed at *δ*_H_ 3.37 (m, H-11a), 3.04 (m, H-11b), 3.12 (m, H-12a) and 2.76 (m, H-12b). Additional methyl resonances were detected at *δ*_H_ 1.97 (s, 16-Me) and 3.80 (s, 8-OMe). Three exchangeable protons assigned to hydroxyl groups were observed at *δ*_H_ 4.09 (d, *J* = 4.8 Hz, 3-OH), 7.30 (s, 9-OH) and 7.78 (s, 15-OH). The ^13^C NMR data (Table 1) further supported the proposed racemosol-type skeleton, with the only difference being the presence of a methoxy substituent. Indeed, based on this evidence, this compound has not been previously reported.

**Figure 3:**
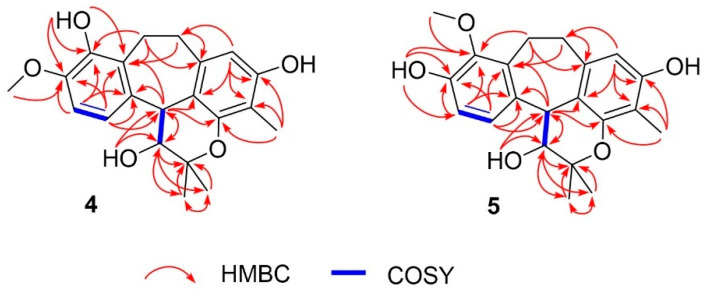
^1^H-^1^H COSY and full HMBC correlations of **4** and **5**.

2D NMR, including ^1^H–^1^H COSY and HMBC data, confirmed the planar structure (Figure 3). The COSY spectrum revealed three spin systems: H-3/H-4, H-6/H-7, and H_2_-11/H_2_-12. The HMBC correlations (Figure 3) provided crucial evidence for assigning the position of the methoxy group. The protons H-6, and H-7 showed correlations to *δ*_C_ 146.5 (C-8), which was also correlated with *δ*_H_ 3.80 (s), corresponding to the methoxy group. Therefore, the methoxy substituent was assigned to C-8 rather than C-9. Other HMBC correlations not mentioned in the text can be seen in Figure 3, which further support this structural assignment.

Since the signals of H-3 and H-4 were overlapped, the NOESY spectrum did not provide sufficient information to determine the relative configuration of the pyran ring. To resolve this issue, the relative configuration of compound **4** was investigated using the DP4+ probability method [11]. The calculated ^13^C NMR chemical shifts of two possible diastereoisomers, **4a** (*cis*) and **4b** (*trans*) (Figure S14), were compared with the experimental data. The DP4+ statistical analysis clearly indicated that compound **4** corresponded to **4a** with 99.59% probability. The absolute configuration of **4** was subsequently determined by ECD. Theoretical ECD spectra for the two enantiomers of **4a** were simulated and compared with the experimental ECD spectrum (Figure 2B). The computed spectrum for the 3*R*, 4*R* absolute configuration showed excellent agreement with the experimental results, thereby confirming the stereochemistry. Consequently, the structure of compound **4** was established as shown in Figure 1.

3-Hydroxy-9-*O*-methylracemosol (**5**) was obtained as a whitish amorphous solid, had the same molecular formula C_21_H_24_O_5_ as compound **4** obtained by the HRESIMS data. The ^1^H and ^13^C NMR data of compound **5** (Table 1) were generally similar to those of compound **4**. However, the presence of a distinct hydroxyl proton signal in the ^1^H NMR spectrum suggested a difference in the position of the methoxy substituent within the same skeleton. The HMBC correlations (Figure 3) provided crucial evidence for determining the position of the methoxy substituent, particularly through the correlations of the aromatic protons. Among them, only proton H-7 (*δ*_H_ 7.04) exhibited a correlation with δ_C_ 145.4 (C-9), which also showed a correlation with *δ*_H_ 3.78, corresponding to the methoxy group. In addition, protons H-11 (*δ*_H_ 3.25 and 3.15) and H-12 (*δ*_H_ 3.17 and 2.80) displayed correlations to C-9 that further supported this substitution pattern.

The absolute configuration of compound **5** was determined by comparison with that of compound **4**, which exhibited an ECD spectrum (Figure 2B) and an optical rotation of the same sign and a comparable magnitude. Compound **5** showed [α]_D_^20^ = +37.3 (*c* 0.10, MeOH), whereas compound **4** showed [α]_D_^20^ = +35.0 (*c* 0.10, MeOH). Based on this similarity, the absolute configuration of **5** was assigned as 3*R*, 4*R*, identical to that of **4**. Likewise, compound **3**, whose absolute configuration had not been previously reported, exhibited [α]_D_^20^ = +43.0 (c 0.10, MeOH) and showed an ECD spectrum with the same Cotton effect sign as compounds **4** and **5** (Figure 2B). Therefore, the absolute configurations of compounds **3**–**5** were assigned accordingly.

The antimicrobial potential of all isolated compounds (**1**–**6**) was evaluated against a full panel of clinically pathogenic Gram-positive and Gram-negative bacteria using the overlay-spot and broth microdilution methods (Table 2 and Table 3).

In the preliminary overlay-spot assay, all compounds demonstrated evident inhibitory activity against Gram-positive species, whereas their effects on Gram-negative organisms were comparatively weaker with the exception of compound **5**. Among the tested isolates, *Bacillus subtilis, Staphylococcus aureus*, *Listeria monocytogenes, Streptococcus pyogenes, and Enterococcus faecalis* exhibited potential in inhibiting bacterial growth following exposure to compounds **1**–**4**, and **6**. In contrast, compound **5** consistently showed no detectable activity across all bacterial strains, suggesting structural specificity among the analogues.

Activity against Gram-negative bacteria was limited, though compounds **1**, **4**, and **6** produced small inhibition against *A. baumannii*, *E. coli* (both ATCC25922 and O157:H7), *K. pneumoniae*, and *S.* Typhi. Notably, only **4** and **6** demonstrated measurable activity against *Pseudomonas aeruginosa* and *Shigella enteritidis*, and these organisms are well known for hospitalized-acquired resistance. These findings highlight the preferential antibacterial spectrum of racemosol and its derivatives toward Gram-positive species. A pattern consistent with other phenolic compounds whose lipophilic character facilitates permeation through the peptidoglycan-rich cell wall of Gram-positive bacteria but is hindered by the outer membrane of Gram-negative bacteria [12,13,14].

Racemosol (**1**) and its active derivatives (**2**, **4**, and **6**) displayed potent activity, with MIC values ranging from 0.156 to 0.625 µg/µL against *S. aureus* ATCC 25923, *B. subtilis*, and *L. monocytogenes*. The corresponding MBCs were generally identical or within a four-fold range of the MICs, indicating a predominantly bactericidal mode of action. Compound **6**, in particular, exhibited the lowest MIC/MBC values (0.156 µg/µL) against several Gram-positive strains, suggesting that structural modifications in this derivative enhance antimicrobial property [15]. Conversely, Gram-negative isolates showed markedly higher MICs (2.5–5 µg/µL) or were non-responsive (MIC > 5 µg/µL). Moderate susceptibility was observed for *A. baumannii* (ATCC 19606) and *E. coli* O157:H7, while *P. aeruginosa* and *S. enteritidis* remained largely resistant. The standard antibiotics ampicillin and gentamicin displayed lower MICs in the microgram, as expected, serving as validation controls for assay performance.

**Figure 4:**
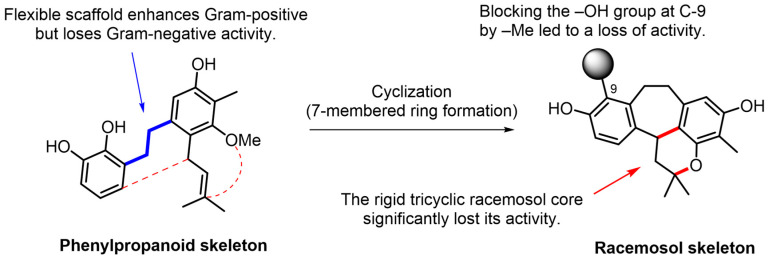
Structure–Activity Relationships (SAR) of racemosol derivatives against bacteria.

The antimicrobial activity of racemosol-type compounds is strongly influenced by their phenolic –OH groups and π-electron delocalization in the aromatic ring [16]. Free –OH groups on the tricyclic racemosol core enable hydrogen bonding with bacterial enzymes and membrane proteins [17,18]. These structures likely enhance solubility and facilitating diffusion through the peptidoglycan layer of Gram-positive bacteria, which explains their potent antimicrobial activity shown in Table 2 and Table 3. However, compound **5** differs from its active analogues by a methyl substitution (R_2_ = Me) on the key phenolic oxygen atom (C-9). This modification eliminates a crucial hydroxyl donor, substantially reducing hydrogen-bonding capacity and increasing lipophilicity, which lowers aqueous solubility and diffusion in culture media. *O*-methylation (–OCH_3_) of key phenols weakens antimicrobial activity in a position-dependent manner, a critical methylation abolishes activity as observed in compound **5**, disrupting both intermolecular binding and intra-molecular H-bond networks [19]. Interestingly, compound **6** contains a phenylpropanoid (C6–C3) scaffold, which is smaller and more flexible than the rigid tricyclic racemosol core found in compounds **1**–**5**. This flexible scaffold enhances activity against Gram-positive bacteria but reduces activity against Gram-negative bacteria. Its flexibility also improves permeability through the Gram-positive peptidoglycan while maintaining sufficient aqueous solubility, resulting in higher effective concentrations at the cell surface and lower MICs. The dual phenolic structure of these compounds allows multi-target engagement via hydrogen-bond donation and acceptance to membrane proteins. The flexible scaffold in compound **6** imposes fewer steric constraints, enabling productive binding to multiple protein pockets, which contributes to its superior antimicrobial activity against pathogenic bacteria [16], as summarized in Figure 4.

## 3. Materials and Methods

### 3.1. General Experimental Procedure

Optical rotations were measured using a JASCO DIP-1000 digital polarimeter (JASCO Inc., Tokyo, Japan). UV and ECD spectra were recorded on a JASCO J-810 spectropolarimeter. IR spectra were obtained using a Bruker Tensor 27 (Bruker, Karlsruhe, Germany). NMR spectra were recorded on a Bruker Avance 400 spectrometer equipped with a cryoprobe (Bruker, Karlsruhe, Germany), using CDCl_3_, or acetone-*d*_6_ as solvents. Residual solvent signals were used as internal references. High-resolution electrospray ionization mass spectrometry (HR-ESI-TOF-MS) data were acquired with a Bruker micrOTOF mass spectrometer (Bruker, Karlsruhe, Germany). Column chromatography was performed on Merck silica gel 60 (230–400 mesh) (Merck, Darmstadt, Germany). Thin-layer chromatography (TLC) was carried out on pre-coated Merck silica gel 60 F_254_ plates (Merck, Darmstadt, Germany). Spots were visualized under UV light (254 and 365 nm) and further detected by spraying with *p*-anisaldehyde reagent followed by heating until charring.

### 3.2. Plant Material

The bark of *B. malabarica* Roxb. was collected from Chum Phae District, Khon Kaen Province, in January 2025. The plant was identified by comparison with an authentic sample, and a voucher specimen (No. AVS-NPR004) has been deposited in the Natural Products Laboratory, Faculty of Applied Science, King Mongkut’s University of Technology North Bangkok, for future reference.

### 3.3. Extraction and Isolation

The MeOH extract (30.0 g) was fractionated by silica gel column chromatography (CC) using a gradient elution of EtOAc in *n*-hexane and EtOAc in MeOH, gradually increasing to 100% MeOH, to afford five fractions, designated BmbA–BmbE. Fraction BmbB was further subjected to CC with an eluent system of 100% CH_2_Cl_2_, gradually increasing to 1:1 (*v*/*v*) EtOAc/CH_2_Cl_2_, yielding six subfractions (BmbB1–BmbB6). Compound **2** (20.0 mg) was isolated from subfraction BmbB4 as a pale-yellow solid by Sephadex LH-20 CC using 100% MeOH as the mobile phase. Further purification of subfraction BmbB6 by CC with acetone/*n*-hexane (2:8, *v*/*v*) afforded compounds **1** (120.0 mg) and **6** (32.0 mg), both obtained as pale-yellow solids. Fraction BmbD was subjected to CC with an eluent system of 100% CH_2_Cl_2_, gradually increasing to 1:1 (*v*/*v*) EtOAc/CH_2_Cl_2_, affording three subfractions (BmbD1–BmbD3). Subfraction BmbD1 was purified by CC with an isocratic eluent system of acetone/n-hexane (3:7, *v*/*v*) to afford compound **5** (10.0 mg) as a pale-yellow solid. Finally, compounds **3** (30.2 mg) and **4** (15.0 mg) were isolated from subfraction BmbD3 as pale-yellow solids via CC with an isocratic eluent system of MeOH/CH_2_Cl_2_ (3:7, *v*/*v*).

3-Hydroxy-8-*O*-methylracemosol (**4**), whitish amorphous solid; UV (MeOH) *λ*_max_ (log *ε*) 212 (2.20), 279 (1.66) nm; ECD (MeOH) λ_max_ (mdeg) 208 (+11.46), 210 (10.02), 220 (+28.94) nm; IR *ν*_max_ 3402, 2924, 2854, 1614, 1517, 1455, 1383, 1249, 1116 cm^−1^; [α]^20^_D_ +35.0 (*c* 0.1, MeOH); ^1^H and ^13^C NMR data, see Table 1; HRESIMS *m*/*z* 355.1549 [M–H]^–^ (calcd for 355.1551, C_21_H_23_O_5_^–^).

3-Hydroxy-9-*O*-methylracemosol (**5**), whitish amorphous solid; UV (MeOH) *λ*_max_ (log *ε*) 213 (2.20), 284 (1.65) nm; ECD (MeOH) λ_max_ (mdeg) 210 (+20.10), 215 (+8.52), 223 (+21.23) nm; IR *ν*_max_ 3382, 2924, 2854, 1614, 1516, 1455, 1374, 1245, 1113 cm^−1^; [α]^20^_D_ +37.3 (*c* 0.1, MeOH); ^1^H and ^13^C NMR data, see Table 1; HRESIMS *m*/*z* 355.1566 [M–H]^–^ (calcd for 355.1551, C_21_H_23_O_5_^–^).

### 3.4. ECD Calculations

All quantum chemical calculations were carried out using the GAUSSIAN 09 software package [20]. Conformational searches were initially performed with the Monte Carlo algorithm employing molecular mechanics force fields implemented in HyperChem Professional 8.0.7 (Hypercube, Inc., Gainesville, FL, USA). The resulting low-energy conformers were subsequently optimized using density functional theory (DFT) at the B3LYP/6-31 + G(d,p) level. Electronic circular dichroism (ECD) spectra were simulated with time-dependent DFT (TD-DFT) calculations at the B3LYP/6-31G(d,p) level using the polarizable continuum model (PCM, σ = 0.50). The theoretical ECD curves were generated using SpecDis 1.64 (University of Würzburg, Germany). For the DP4+ NMR analysis, isotropic magnetic shielding tensors were computed using the gauge-independent atomic orbital (GIAO) method at the mPW1PW91/6-311 + G(d,p) level with the CPCM solvent model. The ^13^C NMR chemical shifts (δ^X^_calc_) were derived from the Boltzmann-averaged shielding tensors according to the equation:

δ^X^_calc_ = σ° − σ_X

The resulting chemical shift data were used to perform the DP4+ probability analysis using the Excel-based DP4+ calculator developed by Grimblat et al. (2015) [11].

### 3.5. Antimicrobial Properties

#### 3.5.1. Determination of Antimicrobial Activity

All bacterial strains utilized in this work were obtained from the Department of Microbiology, School of Medicine, Walailak University, Thailand. The extracts were evaluated against a panel of Gram-positive bacteria, including *Bacillus subtilis* 7988 (clinical isolate), *Bacillus subtilis* ATCC6051, *Enterococcus faecalis* 4232 (Clinical isolates), *Listeria monocytogenes* (clinical isolate), *Staphylococcus aureus* ATCC25923, *Staphylococcus aureus* ATCC29213, methicillin-resistant *Staphylococcus aureus* (MRSA, clinical isolate), *Staphylococcus epidermidis* ATCC35984, and *Streptococcus pyogenes* ATCC19615. The Gram-negative organisms used for antimicrobial screening comprised *Acinetobacter baumannii* ATCC19606, *Acinetobacter baumannii* (MDR strain), *Escherichia coli* ATCC25922, *Escherichia coli* O157:H7, *Klebsiella pneumoniae* ATCC70063, *Pseudomonas aeruginosa* ATCC27853, *Salmonella enterica* serovar Typhi (clinical isolate), and *Shigella enteritidis* (clinical isolate).

All Gram-positive and Gram-negative strains were maintained at 4 °C on nutrient agar or Luria-Bertani (LB) agar (Gibco; Thermo Fisher Scientific, Inc.), respectively. Overnight cultures were prepared in Mueller–Hinton (MH) broth (Gibco; Thermo Fisher Scientific, Inc.) at 37 °C, and subsequently adjusted to approximately 8 × 10^8^ CFU/mL for use in antimicrobial testing. Ampicillin served as the reference antibiotics for Gram-positive bacteria, while gentamicin was used as a standard control for both Gram- negative strains.

#### 3.5.2. Overlay Spot Assay for Antimicrobial Screening

Antimicrobial screening was performed following a modified protocol of Sirisarn et al. (2024) [21]. Briefly, bacterial cultures grown overnight in MH broth at 37 °C were diluted to 10^7^ CFU/mL and spread onto MH agar plates in three directions to achieve uniform lawns. Racemosol and its derivatives were prepared at 10 mg/mL in DMSO and sterilized with a 0.22 μm PTFE filter. Subsequently, 10 μL of each sample was spotted onto the inoculated plates, which were incubated at 37 °C in a 5% CO_2_ atmosphere overnight. The presence of clear inhibition zones on the bacterial lawn was recorded as an indication for antimicrobial activity.

#### 3.5.3. Microdilution Method for MIC and MBC

The broth microdilution procedure for determining minimum inhibitory concentration (MIC) and minimum bactericidal concentration (MBC) was adapted from Sirisarn et al. (2024) [21]. For MIC determination, 100 μL of MH broth was dispensed into each well of a sterile 96-well microplate. Samples were initially diluted to 10 mg/mL in MH broth and subjected to two-fold serial dilutions across the wells, producing concentrations ranging from 5 mg/mL down to 0.00976 mg/mL. To each well, 100 μL of bacterial suspension (10^6^ CFU/mL) was added. As a growth control, 1% DMSO in MH broth was included in parallel. Plates were incubated at 37 °C under 5% CO_2_, and the MIC was defined as the lowest compound concentration that produced complete inhibition, evident by visual clarity of the well contents.

To establish the MBC, 10 μL aliquots from wells at and above the MIC were inoculated onto MH agar plates and incubated at 37 °C in 5% CO_2_. The MBC was defined as the lowest concentration of compound that resulted in the absence of visible bacterial colonies.

## 4. Conclusions

In summary, we isolated four known compounds (**1**–**3**, and **6**) and two new racemosol derivatives (**4** and **5**) from the bark of *B. malabarica*. The structures of the new compounds were elucidated through extensive spectroscopic analysis, and their absolute configurations were established by comparing experimental and calculated ECD curves. Furthermore, GIAO NMR chemical shift calculations, supported by the DP4+ statistical method, were employed to confirm the relative configurations. All isolated compounds were also evaluated for their antibacterial activity against several bacterial strains.

## Figures and Tables

**Table 1:** ^1^H (400 MHz, in ppm, *J* in Hz) and ^13^C NMR (400 MHz) data of compounds **4**–**5** in acetone-*d*_6_.

No.	3-Hydroxy-8-*O*-Methylracemosol (4)		3-Hydroxy-9-*O*-Methylracemosol (5)	
	*δ*_C_	*δ*_H_ (Mult, *J* in Hz)	*δ*_C_	*δ*_H_ (Mult, *J* in Hz)
2	76.9	-	76.9	-
3	71.9	4.31 (m)	71.9	4.28 (m)
4	41.4	4.31 (m)	41.3	4.28 (m)
gem-Me	27.5	1.55 (s)	27.5	1.55 (s)
	17.7	1.23 (s)	17.7	1.23 (s)
4a	115.7	-	115.5	-
5	135.5	-	134.1	-
6	116.0	6.90 (d, 8.6)	114.1	6.68 (d, 8.4)
7	109.0	6.72 (d, 8.6)	121.3	7.04 (d, 8.4)
8	146.5	-	149.2	-
9	143.5	-	145.4	-
10	129.6	-	137.2	-
11	22.3	3.37 (m) 3.04 (m)	23.1	3.25 (m) 3.15 (m)
12	34.3	3.12 (m) 2.76 (m)	34.9	3.17 (m) 2.80 (m)
13	136.4	-	136.1	-
14	110.7	6.11 (s)	110.7	6.11 (s)
15	154.7	-	154.7	-
16	110.1	-	110.2	-
17	152.1	-	152.1	-
16-Me	8.7	1.97 (s)	8.7	1.97 (s)
8-OMe	56.3	3.80 (s)	-	-
9-OMe	-	-	61.3	3.78 (s)
3-OH	-	4.09 (d, 4.8)	-	4.17 (s)
8-OH	-	-	-	7.87 (s)
9-OH	-	7.30 (s)	-	-
15-OH	-	7.78 (s)	-	7.80 (s)

**Table 2:** Antimicrobial activity using overlay spotted assay of compounds **1**–**6** screened against various pathogenic bacteria.

Pathogenic Isolates	Antimicrobial Activity of Compounds 1–6 (100 ug)						Positive Control (amp/gen)	Negative Control (1% DMSO)
	1	2	3	4	5	6		
Gram-positive bacteria								
*Bacillus subtilis* 7988 (Clinical isolate)	+	+	+	+	−	+	+ *	−
*Bacillus subtilis* ATCC6051	+	+	+	+	−	+	+ *	−
*Enterococcus faecalis* 4232 (Clinical isolates)	+	+	+	+	−	+	+ *	−
*Listeria monocytogenes* (Clinical isolates)	+	+	+	+	−	+	+ *	−
*Staphylococcus aureus* ATCC25923	+	+	+	+	−	+	+ *	−
*Staphylococcus aureus* ATCC29213	+	+	+	+	−	+	+ *	−
Methicillin Resistant *Staphylococcus aureus* (Clinical isolate)	+	+	+	+	−	+	− *	−
*Staphylococcus epidermitis* 35984	+	+	+	+	−	+	+ *	−
*Streptococcus pyogenes* ATCC49619	+	+	+	+	−	+	+ *	−
Gram-negative bacteria					−			−
*Acinetobacter baumannii* ATCC19606	+	−	−	+	−	+	+ **	−
Multidrug-resistant *Acinetobacter baumannii* (MDR)	+	+	−	+	−	+	− **	−
*Escherichia coli* ATCC25922	+	+	−	+	−	+	+ **	−
*Escherichia coli* O157:H7	+	+	+	+	−	+	+ **	−
*Klebsiella pneumoniae* ATCC70063	+	+	−	+	−	+	+ **	−
*Pseudomonas aeruginosa* ATCC27853	−	−	−	+	−	+	+ **	−
*Salmonella enterica* serotype Typhi (Clinical isolates)	+	+	−	+	−	+	+ **	−
*Shigella enteritis* (Clinical isolates)	−	−	−	−	−	−	+ **	−

10 µg/mL of ampicillin (amp) was used as positive control for Gram-positive bacteria marked as * and 10 µg/mL of gentamicin (gen) was used as positive control for Gram-negative bacteria marked as **. DMSO was used as a negative control. Symbol + and − stand for making a clear spot and not making a clear spot on bacterial lawn, respectively. All tests were conducted in triplicate.

**Table 3:** Minimum inhibitory concentrations and minimum bactericidal concentrations of compounds **1**–**6** against various pathogenic bacteria.

Pathogenic Isolates	Minimum Inhibitory Concentration (MIC)/Minimum Bactericidal Concentration (MBC) in µg/µL of Compounds 1–6						Minimum Inhibitory Concentration (MIC) of Ampicillin/Gentamycin (µg/mL)	Negative Control (1% DMSO)
	1	2	3	4	5	6		
Gram-positive bacteria								
*Bacillus subtilis* 7988 (Clinical isolate)	0.625/0.625	0.625/2.5	2.5/2.5	0.625/0.625	NT	0.15625/0.15625	0.5/0.5	R
*Bacillus subtilis* ATCC6051	0.15625/>5	0.3125/>5	1.25/>5	0.3125/>5	NT	0.15625/>5	0.125/0.125	R
*Enterococcus faecalis* 4232 (Clinical isolates)	0.15625/>5	0.625/>5	2.5/>5	1.25/>5	NT	0.3125/0.625	0.25/NA	R
*Listeria monocytogenes* (Clinical isolates)	0.15625/0.3125	0.625/>5	0.625/>5	0.3125/1.25	NT	0.3125/0.3125	0.25/2	R
*Staphylococcus aureus* ATCC25923	0.15625/0.3125	0.625/>5	0.625/>5	0.625/>5	NT	0.3125/0.3125	0.25/0.5	R
*Staphylococcus aureus* ATCC29213	0.15625/0.3125	0.625/>5	0.625/>5	0.3125/>5	NT	0.3125/0.625	0.25/0.125	R
Methicillin Resistant *Staphylococcus aureus* (Clinical isolate)	0.15625/0.15625	0.625/>5	0.625/>5	0.3125/>5	NT	0.3125/0.3125	NA/0.25	R
*Staphylococcus epidermitis* 35984	0.15625/0.625	1.25/>5	1.25/>5	0.15625/>5	NT	0.3125/0.625	0.5/0.25	R
*Streptococcus pyogenes* ATCC49619	0.3125/0.3125	1.25/>5	1.25/>5	0.3125/>5	NT	0.3125/0.625	0.25/0.125	R
Gram-negative bacteria								
*Acinetobacter baumannii* ATCC19606	0.3125/0.625	NT	NT	0.3125/5	NT	0.15625/0.3125	NT/1	R
Multidrug-resistant *Acinetobacter baumannii* (MDR)	1.25/5	1.25/>5	NT	1.25/5	NT	1.25/5	NT/NA	R
*Escherichia coli* ATCC25922	2.5/>5	2.5/>5	NT	2.5/5	NT	2.5/>5	NT/0.031	R
*Escherichia coli* O157:H7	2.5/5	2.5/5	2.5/5	2.5/>5	NT	2.5/5	NT/0.063	R
*Klebsiella pneumoniae* ATCC70063	2.5/>5	2.5/>5	NT	2.5/>5	NT	2.5/>5	NT/0.062	R
*Pseudomonas aeruginosa* ATCC27853	NT	NT	NT	2.5/>5	NT	2.5/5	NT/4	R
*Salmonella enterica* serotype Typhi (Clinical isolates)	2.5/>5	2.5/>5	NT	2.5/>5	NT	2.5/5	NT/2	R
*Shigella enteritis* (Clinical isolates)	NT	NT	NT	NT	NT	NT	NT/1	R

Ampicillin (amp) was used as positive control for Gram-positive bacteria and Gentamycin (gen) was used as positive control for both Gram-positive and Gram-negative bacteria. DMSO (1%) was used as a negative control diluted by MHB broth. The letter R denotes resistance, a parameter that was not employed in the investigation of minimum inhibitory concentration (MIC). NA indicates “not active,” meaning no antibacterial activity was observed at the tested concentrations. NT indicates “not testing” meaning no MIC and MBC was carried out due to the lack of clear spot area from overlay spotted assay.

## Data Availability

The data presented in this study are available in the present article and the Supplementary Material.

## Supplementary Materials

The following supporting information can be downloaded at: https://www.mdpi.com/article/10.3390/molecules30214308/s1, 1D and 2D NMR spectra of compounds **4** and **5**.
