# Supplementary material for: Racemosol Derivatives and Other Metabolites from Bauhinia malabarica Bark with Antibacterial Activity
Source: Molecules. 2025 Nov 5;30(21):4308. doi: 10.3390/molecules30214308 (PMC12608365; doi:10.3390/molecules30214308)
Supplement: Supplementary file 1 [file molecules-30-04308-s001.zip › molecules-3942379-supplementary.pdf]

Racemosol Derivatives and Other Metabolites from *Bauhinia malabarica* Bark with Antibacterial Activity

Wanchat Sirisarn [1], Apisara Somteds [2], Supachai Jadsadajerm [2], Sutin Kaennakam [3], Nuttapon Yodsin [4] and Awat Wisetsai [2,*]

[1] Department of Microbiology, Faculty of Medicine, Kasetsart University, Bangkok 10900, Thailand; wanchat.s@ku.th

[2] Department of Industrial Chemistry, Faculty of Applied Science, King Mongkut's University of Technology North Bangkok, Bangkok 10800, Thailand; somteds.apisara@gmail.com (A.S.); supachai.j@sci.kmutnb.ac.th (S.J.)

[3] Department of Agro-Industrial, Food, and Environmental Technology, Faculty of Applied Science, King Mongkut's University of Technology North Bangkok (KMUTNB), Bangkok 10800, Thailand; sutin.k@sci.kmutnb.ac.th

[4] Department of Chemistry, Faculty of Science, Silpakorn University, Nakorn Pathom 73000, Thailand; yodsin_n@su.ac.th

* Correspondence: awat.w@sci.kmutnb.ac.th

Content

		Page
**Figure S1.**	HR-ESIMS spectrum of **4**.	3
**Figure S2.**	^{1}H NMR spectrum (400 MHz, acetone-d6) of **4**.	3
**Figure S3.**	^{13}C NMR spectrum (100 MHz, acetone-d6) of **4**.	4
**Figure S4.**	COSY spectrum of **4**.	4
**Figure S5.**	HSQC spectrum of **4**.	5
**Figure S6.**	HMBC spectrum of **4**.	5
**Figure S7.**	NOSY spectrum of **4**.	6
**Figure S8.**	HR-ESIMS spectrum of **5**.	6
**Figure S9.**	^{1}H NMR spectrum (400 MHz, acetone-d6) of **5**.	7
**Figure S10.**	^{13}C NMR spectrum (100 MHz, acetone-d6) of **5**.	7
**Figure S11.**	COSY spectrum of **5**.	8
**Figure S12.**	HSQC spectrum of **5**.	8
**Figure S13.**	HMBC spectrum of **5**.	9
**Figure S14.**	DP4+ analysis of isomer 1 (**4a**) and isomer 2 (**4b**) of **4**.	9

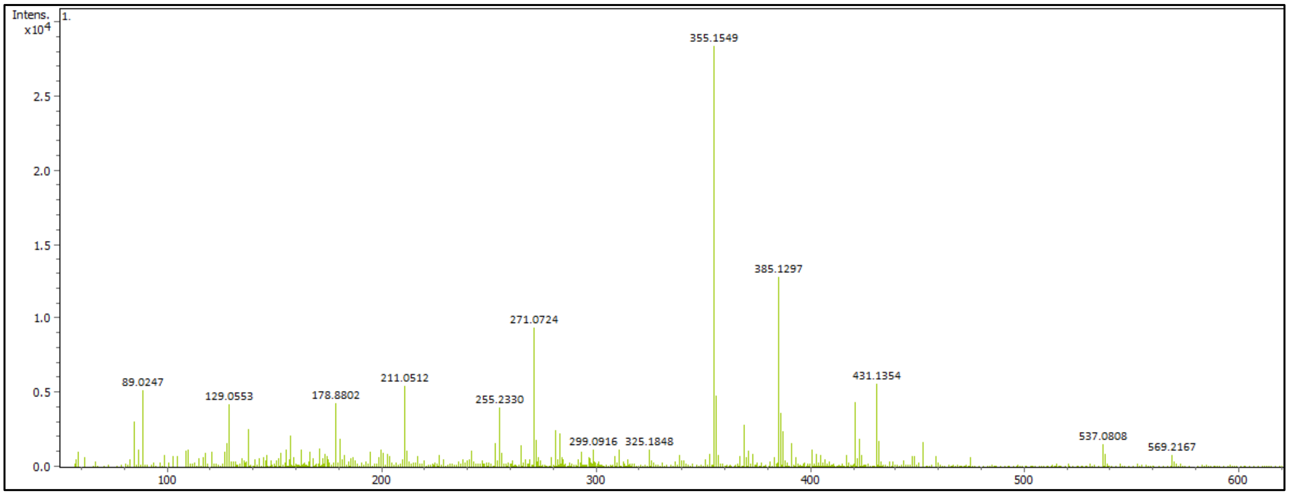

Figure S1. HR-ESIMS spectrum of **4**.

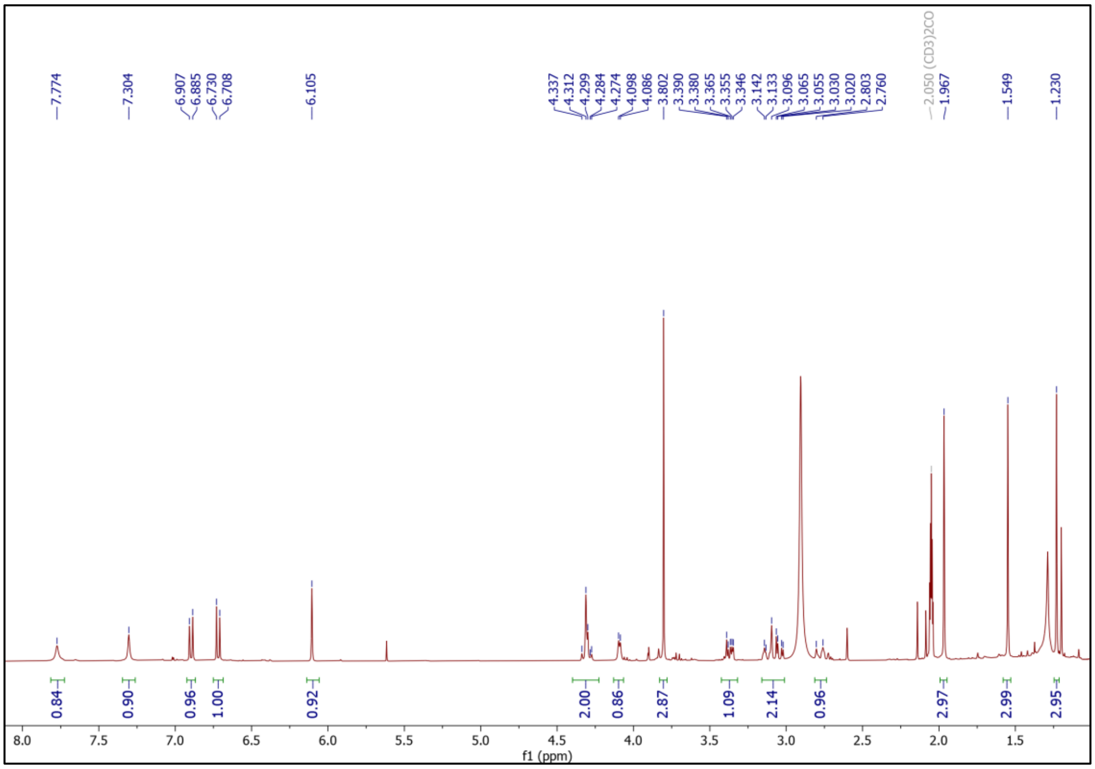

Figure S2. ^{1}H NMR spectrum (400 MHz, acetone-d6) of **4**.

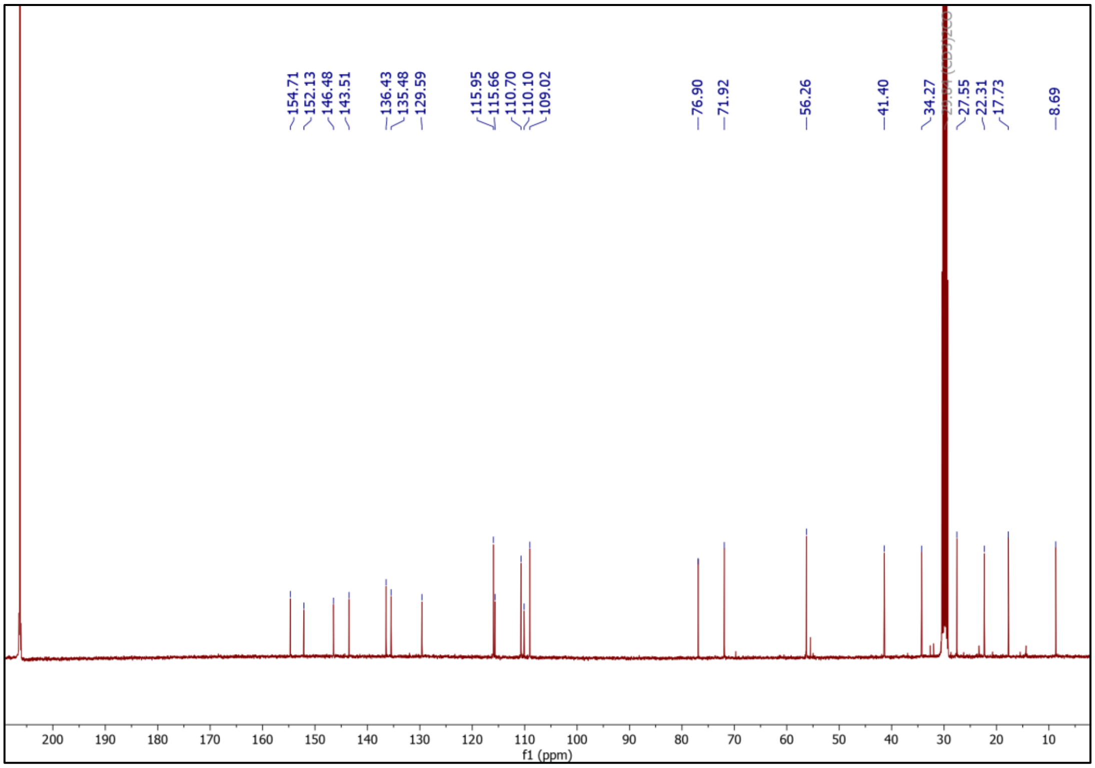

Figure S3. ^{13}C NMR spectrum (100 MHz, acetone-d6) of **4**.

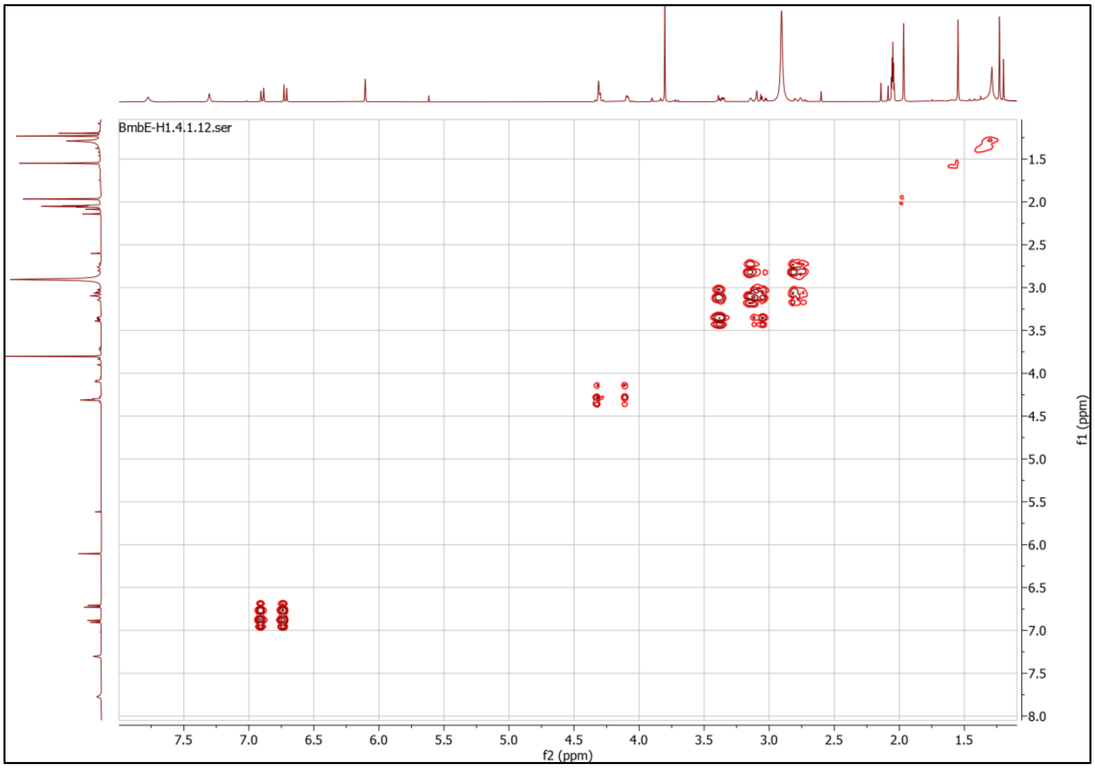

Figure S4. COSY spectrum of **4**.

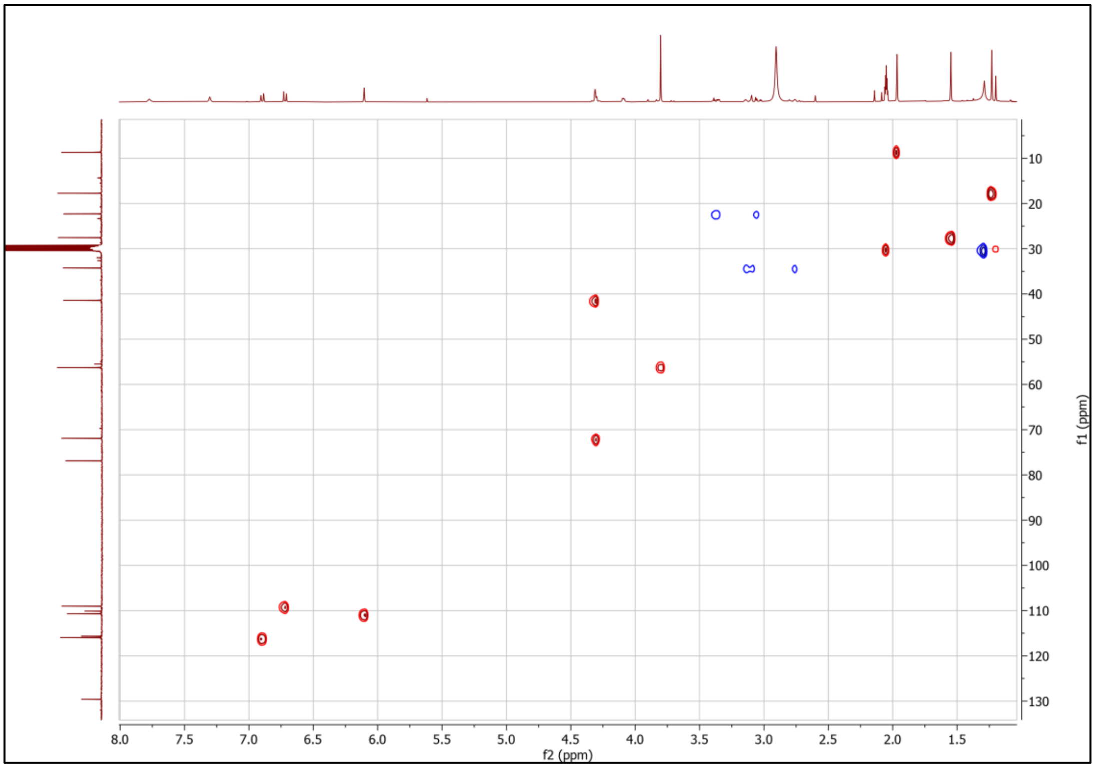

Figure S5. HSQC spectrum of **4**.

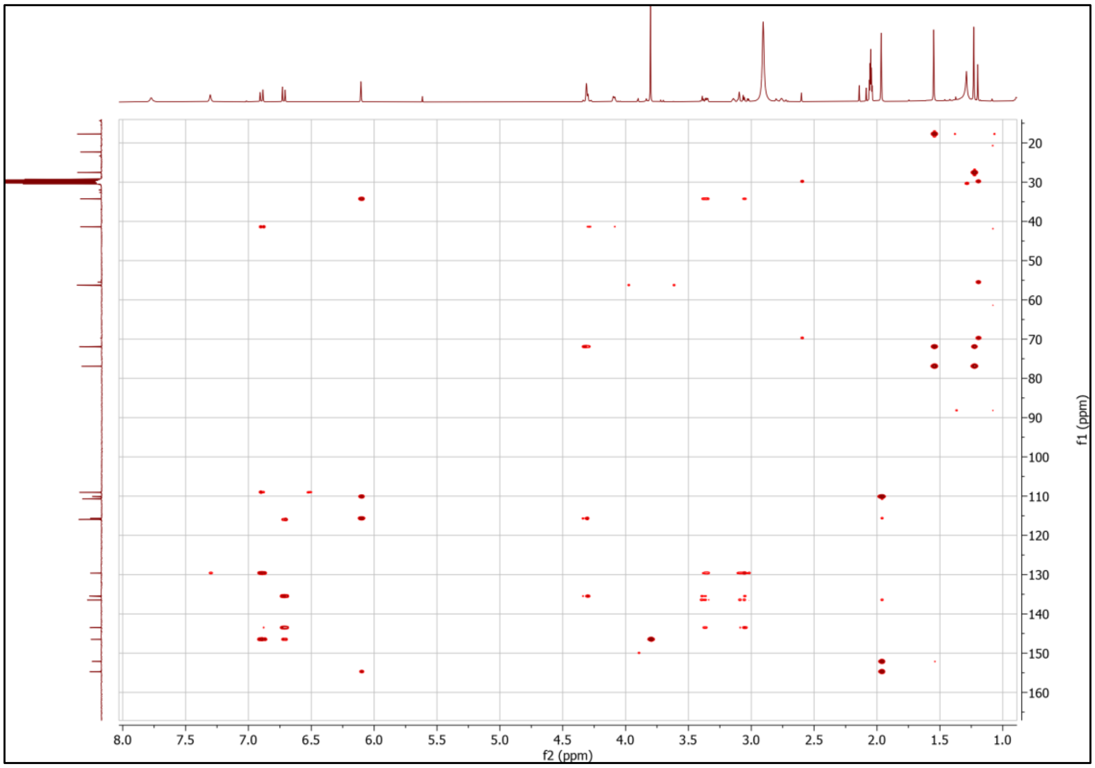

Figure S6. HMBC spectrum of **4**.

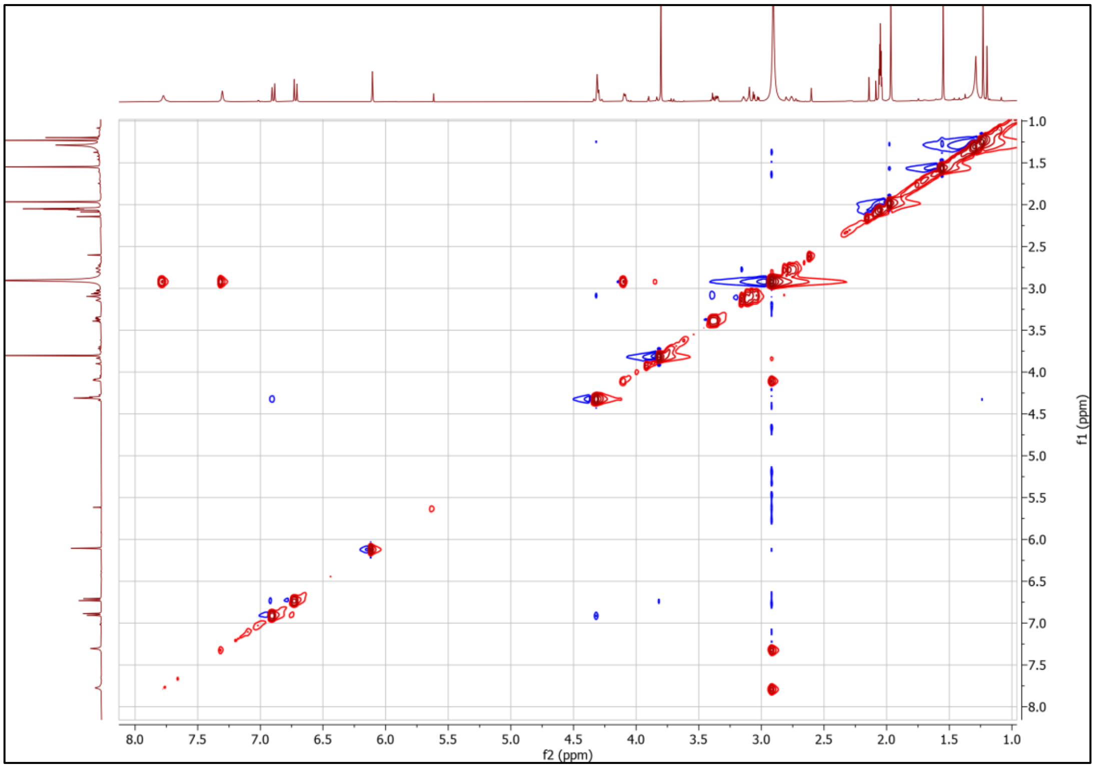

Figure S7. NOSY spectrum of **4**.

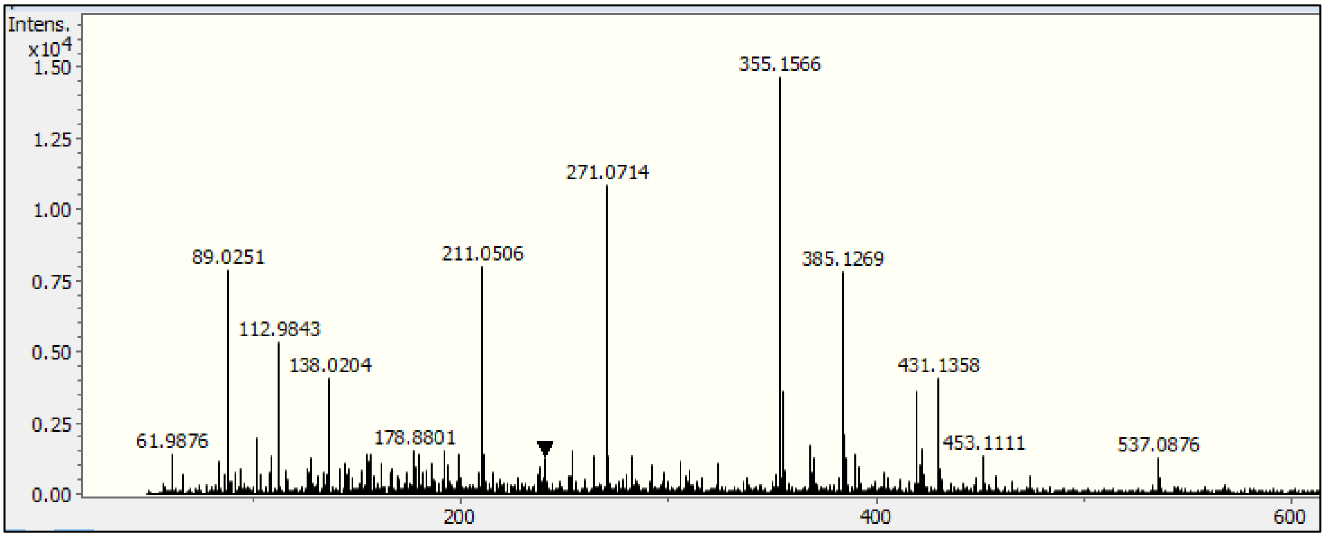

Figure S8. HR-ESIMS spectrum of **5**.

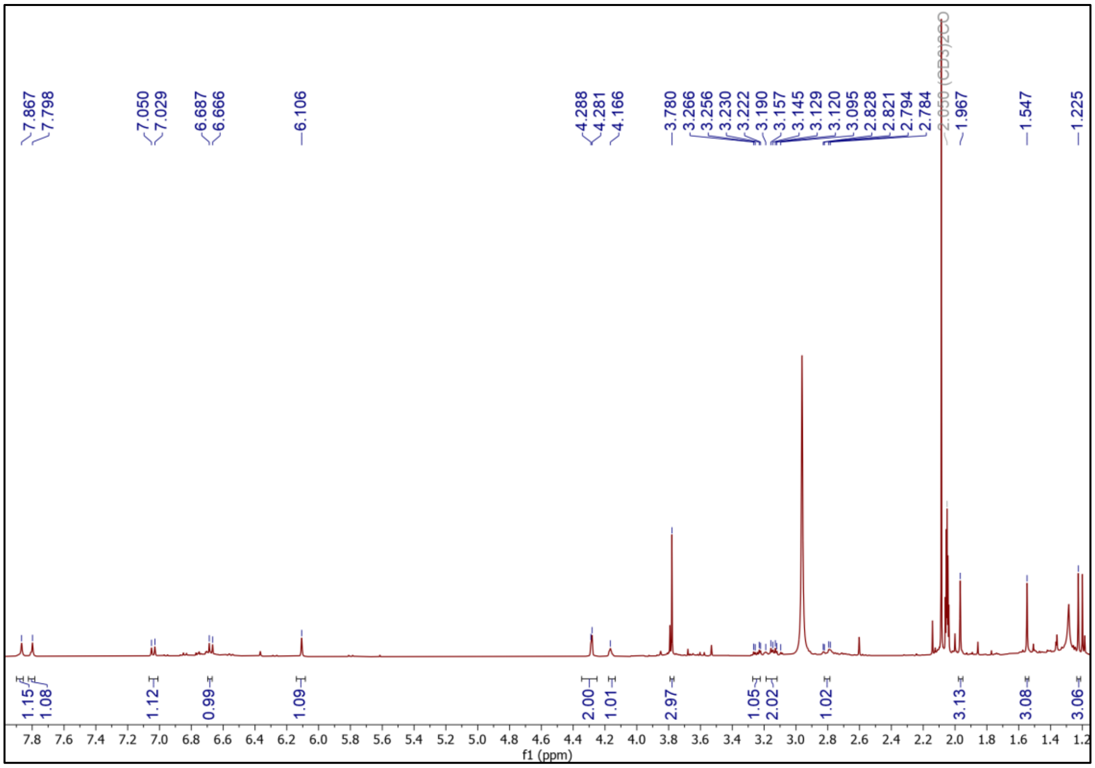

Figure S9. ^{1}H NMR spectrum (400 MHz, acetone-d6) of **5**.

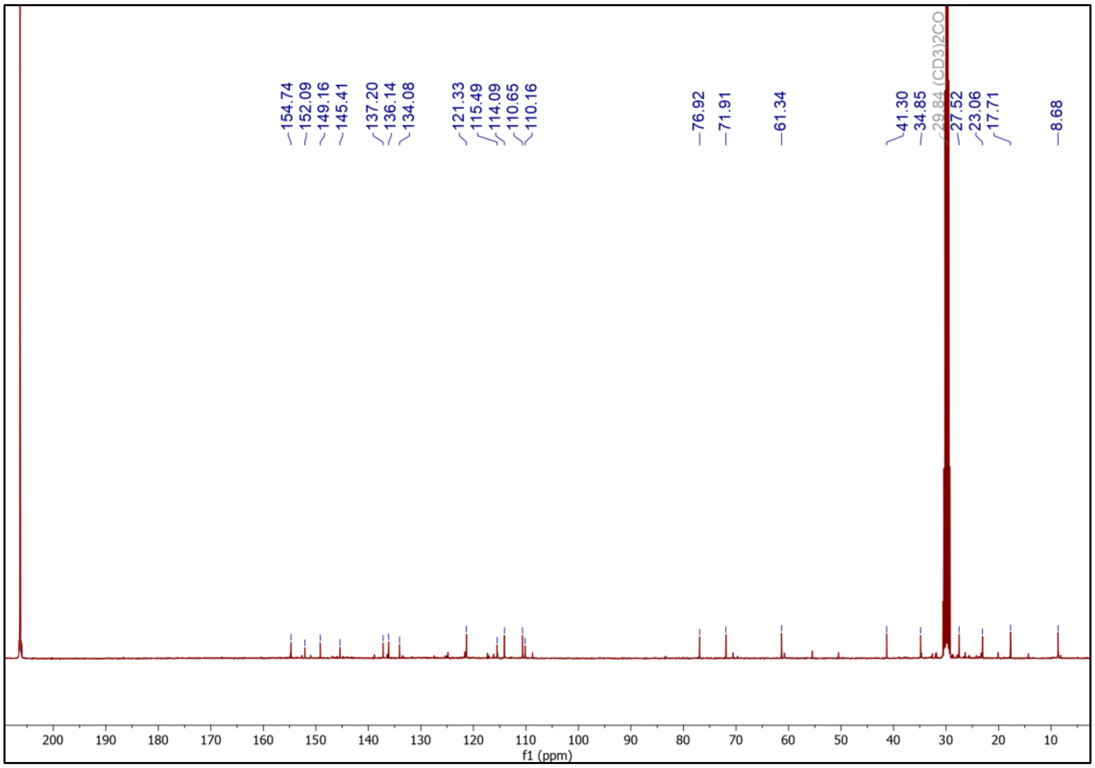

Figure S10. ^{13}C NMR spectrum (100 MHz, acetone-d6) of **5**.

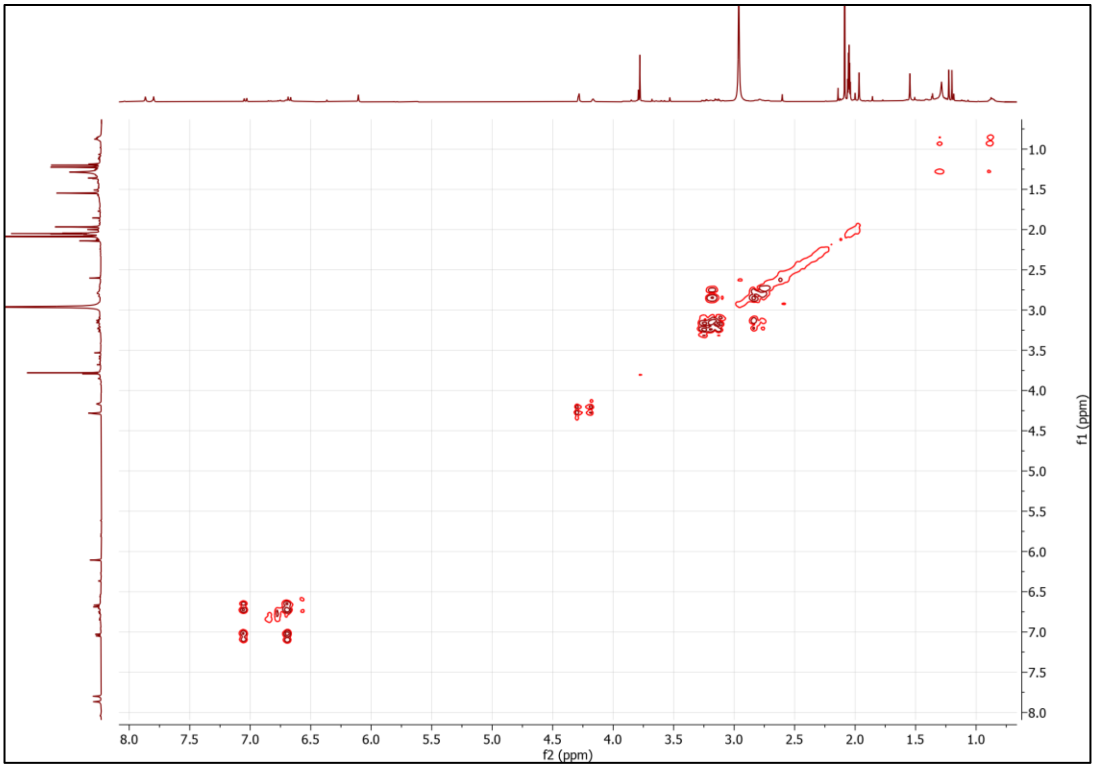

Figure S11. COSY spectrum of **5**.

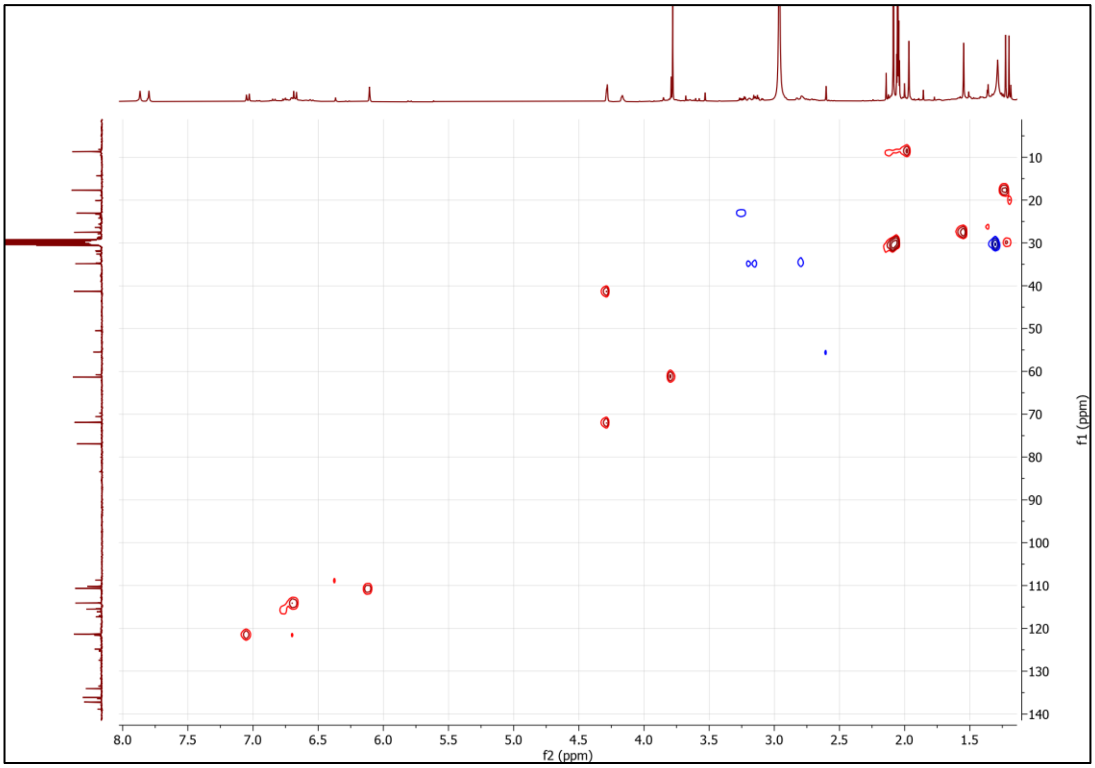

Figure S12. HSQC spectrum of **5**.

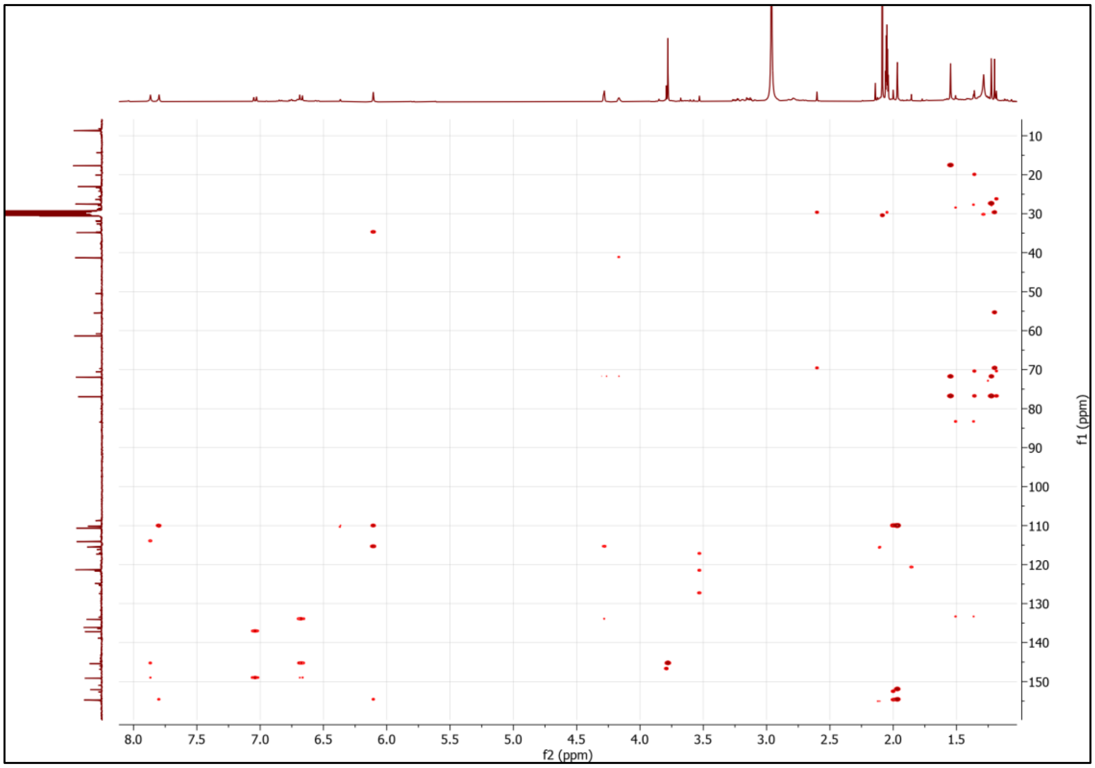

Figure S13. HMBC spectrum of **5**.

Functional	Solvent?	Basis Set	Type of Data
mPW1PW91	PCM	6-31+G(d,p)	Shielding Tensors

		DP4+	99.59%	0.41%	-	-	-
**Nuclei**	**sp2?**	**Experimental**	**Isomer 1**	**Isomer 2**	**Isomer 3**	**Isomer 4**	**Isomer 5**
c		154.7	156.6	156.6			
c		152.1	153.5	152.1			
c		146.5	150.9	150.7			
c		143.5	147.3	147.5			
c		136.4	143.8	139.7			
c	x	135.5	134.5	134.7			
c	x	129.6	130.1	124.0			
c	x	116	122.3	122.0			
c	x	115.7	118.5	118.7			
c	x	110.7	111.2	115.8			
c	x	110.1	106.9	107.7			
c	x	109	104.55	105.78			
c		76.9	74.34	76.18			
c		71.9	68.82	72.88			
c	x	56.3	54.30	54.23			
c	x	41.4	40.01	41.58			
c	x	34.3	31.51	29.09			
c	x	27.5	25.98	24.85			
c	x	22.3	21.42	22.63			
c		17.7	17.18	12.34			
c		8.7	2.09	1.88			

Functional	Solvent?	Basis Set	Type of Data
mPW1PW91	PCM	6-31+G(d,p)	Shielding Tensors

	Isomer 1	Isomer 2	Isomer 3	Isomer 4	Isomer 5	Isomer 6
sDP4+ (H data)	-	-	-	-	-	-
sDP4+ (C data)	99.59%	0.41%	-	-	-	-
sDP4+ (all data)	99.59%	0.41%	-	-	-	-
uDP4+ (H data)	-	-	-	-	-	-
uDP4+ (C data)	50.07%	49.93%	-	-	-	-
uDP4+ (all data)	50.07%	49.93%	-	-	-	-
DP4+ (H data)	-	-	-	-	-	-
DP4+ (C data)	99.59%	0.41%	-	-	-	-
DP4+ (all data)	99.59%	0.41%	-	-	-	-

Isomer 4a (*cis*) **Isomer 4b (*trans*)**

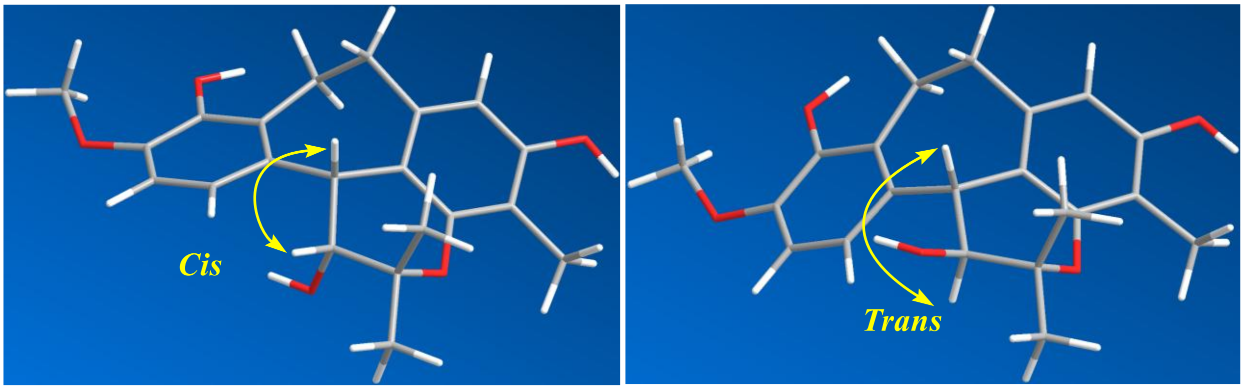

Figure S14. DP4+ analysis of isomer 1 (**4a**) and isomer 2 (**4b**) of **4**.
